# Bioengineering and computational analysis of programmed cell death ligand-1 monoclonal antibody

**DOI:** 10.3389/fimmu.2022.1012499

**Published:** 2022-10-21

**Authors:** Muhammad Kalim, Hamid Ali, Ashfaq Ur Rehman, Yong Lu, Jinbiao Zhan

**Affiliations:** ^1^ Department of Biochemistry and Cancer Institute of the Second Affiliated Hospital, Zhejiang University, School of Medicine, Hangzhou, China; ^2^ Department of Biosciences, COMSATS University, Islamabad, Pakistan; ^3^ Department of Molecular Biology and Biochemistry, University of California, Irvine, Irvine, CA, United States; ^4^ Laboratory of Minigene Pharmacy, School of Life Science and Technology, China Pharmaceutical University, Tongjia Xiang, Nanjing, China

**Keywords:** PD-L1, recombinant technology, monoclonal antibody, protein-protein interaction, chimera

## Abstract

The trans-membrane proteins of the B7 family programmed cell death ligand-1 (PD-L1) and programmed death-1 (PD-1) play important roles in inhibiting immune responses and enhancing self-tolerance *via* T-cell modulation. Several therapeutic antibodies are used to promote T-cell proliferation by preventing interactions between PD-1/PD-L1. Recombinant technology appears to be quite useful in the production of such potent antibodies. In this study, we constructed recombinant molecules by cloning variable regions of the PD-L1 molecule into pMH3 vectors and transferring them into mammalian cell lines for expression. G418 supplementation was used to screen the recombinant clones, which were then maintained on serum-free medium. The full-length antibody was isolated and purified from the medium supernatant at a concentration of 0.5-0.8 mg/ml. Antibody binding affinity was investigated using ELISA and immunofluorescence methods. The protein-protein interactions (PPI) were determined using a docking approach. The SWISS model was utilized for homology modeling, while ZDOCK, Chimera, and PyMOL were used to validate 3D models. The Ramachandran plots were constructed using the SWISS model, which revealed that high-quality structures had a value of more than 90%. Current technologies allow for the accurate determination of antigen-antibody interactions.

## Highlights

1. Recombinant antibody production provides an alternative to classical polyclonal antibody production.
2. The full-length antibody was optimized using CHO host cell machinery.
3. PPI serves as a foundation for understanding cellular biological and molecular processes.
4. Anti-PD-L1 describes the ability to bind the target antigen.

## Introduction

Programmed cell death ligand-1 (PD-L1) is a 40kDa trans-membrane protein of the B7 family that shares 40% homology with B7-DC/PD-L2 recorded more homologous to one another in this group (1, 2). By reducing the secretion of interleukin-10, IL-4, and IL-2 as well as the generation of interferon through association with PD-1 receptors, these member interactions result in the downregulation of T cell activation (3, 4). PD-L1 interaction to its receptors B7.1 (CD80) and PD-1 suppresses T cell proliferation, migration, and cytotoxic mediators’ secretion (5, 6). Activated T cell and B cell expresses PD-1 expression while PD-L1 can be induced in macrophages and dendritic immune cells with inflammatory cytokines. The down-regulation can be released by inhibition of PD-L1/PD-1 immune checkpoints *via* antibody therapies (7).

Chimeric antibodies are produced using a variety of expression techniques. Mammalian cells, plant cells, fungus, and bacterial cells make up these host systems. Among all of these mammalian cell lines, Chinese Hamster Ovaries (CHO) has been identified as a suitable host for various therapeutics studies. (8–10). More than 50% of approved antibody production utilizes mammalian cell host machinery. Various studies were reported for optimum expression in a short time to further elucidate its efficient production (11, 12). The ExpiCHO cell expression system became available in 2015 that stabilizes the combination of CHO cell lines, transfection strategies, and maximum production of antibodies.

The monoclonal antibody has emerged as a promising approach for treating immune checkpoint inhibition in numerous malignancies (13–15). These antibodies are more expensive to produce and require genetic maintenance of unstable hybridoma cell cultures. Furthermore, the interaction of Fc domains by immune responses might cause phagocytosis or fixation, which can obstruct research results and interfere with therapeutic benefits. (16–18). It was investigated to create more compact antibody fragments, like scFv, to address issues with full-length antibodies (19). The heavy and light chain variable regions make up the scFv fragments. An effective source of recombinant full-length antibody expression can be obtained from these variable regions, which are connected by a flexible linker (20). Jin, et al., outlined the achievements in cancer therapy of new-format therapeutic antibodies, such as antibody conjugates (e.g., ADCs and radiolabeled antibodies), bispecific/multispecific antibodies, immune cytokines, antibody fragments (e.g., Fabs, scFvs, and VHH domains), and scaffold proteins. Full-length antibodies, such as Fabs, scFvs, and VHH domains, have been transformed into fragments, and small scaffold proteins (e.g., affibodies and DARPins) have been rationally designed to enhance tumor penetration and facilitate fast serum clearance, which are advantages for their applications in tumor diagnostic imaging (21). Over 30 antibody fragment engineering platforms are now producing novel antibody fragment forms for cancer therapy. Because of the increasing number of engineering strategies and formats available for the production of novel antibody medicines, careful selection of a suitable strategy is essential. To create the optimum medication for clinical advantages, the binding affinity, avidity, valency, epitope interaction/accessibility, stability and flexibility, and half-life of the format must all be optimized (22).

Many molecular modeling tools have been used to explore complicated chemical and biological systems in a range of drug or antibody development programs in the current era of pharmaceutical and medical research (23). In the identification, characterization, and development of novel and beneficial therapeutics, it is critical to incorporate experimental procedures into computational methodologies. Molecular docking is a technique used widely in current protein/antibody research that examines the conformations of antibody fragments within the macromolecular target binding site and calculates the receptor-ligand binding free energy for all possible conformations (24). The binding affinity of the complex is determined after small molecular molecules (scFv) are docked into the receptor’s binding site. This is a crucial step in the structure-based medication development process (25). The capacity to view binding interactions and geometries utilizing a quick and precise docking approach is necessary for a complete understanding and estimation of the protein-protein (PP) or antigen-antibody complex (26). A variety of docking approaches are currently accessible, which can allow researchers better understand the benefits and downsides of these techniques. However, the majority of free tools rely on command-line knowledge. For biologists, this is a time-consuming process, therefore they avoid it. A proper estimate of each method can lead to the creation of feasible strategies and reproducible and relevant outcomes.

The current study describes the expression of full-length antibodies against PD-L1 derived from scFv-PDL1 fragments in CHO host cells. ELISA and cell surface binding interaction were used to validate the bioactivity of the chimeric anti-PDL1 in the laboratory. ZDOCK, UCSF Chimera (https://www.cgl.ucsf.edu/chimera/), and the PyMOL (http://pymol.org/2/) docking tools were used to predict PP complex structures. In a serum-free medium, transfection and expression methods were used. The expressed antibody was found to have a high affinity and efficacy toward the PD-L1 surface antigen. The present studies of wet-lab and in-silico studies evaluation can be productive in innovative pharmaceutics development in a variety of cancers by blocking PD-L1/PD-1 interactions.

## Materials and methods

### Reagents, cell lines, and plasmids

Tumor cell lines A549 (Adenocarcinoma, Lung Cancer), BEL-7402 (Hepatic Carcinoma) and MDA453 were obtained from the American Type Culture Collection (ATCC) and were maintained in RPMI-1640 supplemented with 10% fetal bovine serum in a humidified incubator containing 5% CO2 at 37°C. The CHO cells were cultured in DMEM medium supplemented with 10% FBS before transfection. Later on, the transformed CHO cells were maintained in serum-free medium Ex-Cell. Different bacterial strains were maintained under strict sterile conditions. The scFv fragments were engineered previously by our group that comprised a single polypeptide chain with the variable region of heavy and light chains attached by Gly-Ser flexible linker. The fragments were used as a template for polymerization of full-length antibody PD-L1 extracellular domain (27). Anti-PDL1 IgG antibody was from Life Science Production & Services, China, and was used as a control. Rabbit anti-human IgG (H+L)-HRP (Cat No 6140-05; Lot No D2311-ZD51E) were from Southern Biotech, USA and rabbit anti-human IgG-FITC (Cat No HA1001; Lot No G161011) were provided by Hangzhou HunAn Biotech, Comp, China. Plasmid pMH3-kH/kL, free serum Ex-cell CHO media, and CHO cells were gifted by Prof. Dr. Shuqing Chen. All reagents, solutions and buffers were maintained under high-grade purity and strict sterile conditions. All procedures involving call cultures were approved by the Laboratory Animal Care and Committee of Zhejiang University (approval number, ZJU20190038).

### Construction of pMH3-VH/VL recombinant plasmid and PCR amplification

Primers were constructed for local peptide sequence with the addition of *Eco*RI and *Nhe*I in the heavy fragment to facilitate the recombinant SP-VH development. Similarly, the same *Eco*RI and *Bsiw*I were added to signal peptide and VL sequences (**Table 1**). There were no other similar restriction sites found in the internal framework of signal peptides, variable and light chain sequences. Amplification of VH/VL fragments from selected frozen scFv-PDL1 was carried out in a thermocycler reaction mix of 50µl. The condition was settled out as recommended for the DNA Prime star polymerase kit. DNA extracted fragments were loaded on 1% agarose gel and were recovered by a DNA extraction kit (Takkara).

**Table 1:** List of primers used during recombinant development of full-length antibody.

Primer Name	Sequences (5’ to 3’)
**MHF**	CGAATTCCACCATGGAGAAAGACAC
**MHR**	CAAGCTGGACCTGACCAGTGGAAC
**MHF**	CGAATTCCACCATGGAGAAAGACAC
**MVR**	CACCCGGATGTCACCAGTGGAAC
**VHF**	GTTCCACTGGTCAGGTCCAGCTTG
**VHR**	CTAGCTAGCTGAAGAGACAGTGACG
**VLF**	GTTCCACTGGTGACATCCGGGTG
**VLR**	CTACGTACGTTTAATCTCCAGTC

MHF represents forward primer for IgK signal peptide; MHR represents reverse primer for heavy chain; MVR represents reverse primer for variable light chain; VHF represents forward primer for VH region; VHR represents reverse primer for VH region; VLF represents forward primer for VL region; VLR represents reverse primer for VL region.

### Overlap PCR ligation to develop SP-VH/VL fragments

The recovered fragments of the light chain and heavy chain (VH/VL) were joined together with signal peptide sequences (SP) in PCR. The first master mix was prepared separately for both VH and VL fragments, with the addition of 1µl signal peptide and 1µl of VH/VL fragments, 10µl of 5x Prime Star buffer, 0.5µl of Prime Star DNA polymerase and the total volume of 50µl were equilibrated with the addition of ddH_2_O at final. The first PCR conditions were settled out without the addition of primers as 1 cycle denaturation at 94°C for 5 min followed by 8 cycles (denaturation at 94°C for 1 min) and annealing at 58°C, and elongation at 72°C for 1 min) additional elongation of 10 min at 72°C. The second cycle of polymerization was carried out with the addition of forward and reverse primers for a total of 25-30 cycles. The final PCR products were loaded on 1% agarose gel and were resolved by using an extraction kit to purify the product.

### Double digestion of SP-VH/VL and recombinant pMH3-kH/kL vector construction

A double digestion system was created for both heavy and light pMH3 cloning vector along with SP-VH/VL fragment to develop the pMH3-kH/kL recombinant cloning vector. A single digestion cycle was carried out against heavy fragments, facilitated by our designed primer including *Eco*RI and *Nhe*I restriction sites as shown in **Table 1**. Meanwhile, two rounds of digestion cycles were settled out for light chain fragments assisted by *Eco*RI and *Bsiw*I restrictive enzymes. The pMH3- kH/kL was purified from frozen *E. coli* cells. A 20µl mix of purified SP-VH and pMH3-kH fragments were incubated with enzymes at 37°C for 3 hours and recovered by 1% gel. Similarly, SP-VL and pMH3-kL were digested in two cycles separately with the addition of *Eco*RI and *Bsiw*I. The final extract was purified with 1% gel and recovered by a DNA digestion kit. Both products were eventually ligated separately to develop pMH3-kH and pMH3-kL recombinant vectors with the ease of T4 ligase enzymes. The recombinant vector was resolved on 0.8-1% agarose gel and purified by the kit.

### Transformation and sequence analysis

The recombinant pMH3-kH/kL vectors were cloned into competent *E. coli* (DH5) and plated on LB agar plates supplemented with 100µg/ml ampicillin. After overnight incubation at 37°C, the single clone was collected and incubated in 5ml LB broth media. Plasmids were extracted and the integrity of recombinant fragments was confirmed by restriction digestion and polymerization reaction. The aliquots were collected and sent out for sequencing. The final confirmed positive clones were saved and analyzed further for animal cell (CHO) transfection.

### Transfection of CHO cells

The Chinese Hamster Ovary cells were reinvigorated from the stock culture in DMEM medium supplemented with 10% FBS. The cells were maintained for up to two generations and then 1x10^6^ cells were inoculated into 6 wells plate and incubated until 80% confluent. Plasmids were linearized before transfection. 6µl of Lipofectamine 6000 was mixed with 100µl RPMI 1640 without FBS supplements and incubated for 5 minutes. Similarly, recombinant plasmid DNA (3µg/µl) of each VH and VL were added to 100µl RPMI 1640 without FBS and incubated for 5 minutes. The plasmid solutions and Lipofectamine were mixed and incubated for 20 minutes. The final mixture was added dropwise to RPMI 1640 medium without FBS into 6 wells plate of CHO cells. The cells were incubated at 37°C for 6 hours to allow transfection. Cells were washed and 2 ml DMEM supplemented with 10% FBS media was added and incubated for 24 hours. After 24 hours, G418 was added at a final concentration of 700µg/ml for pool selection and incubated for another 10 days. Each day, aliquots were collected and ELISA tests were performed, and transfected cells were tested for G418 activity. Limited dilution was achieved by inoculating a single cell into a 96-well plate and allowing it to incubate for another 10-14 days. Cells were transplanted to 24 wells, 16 wells, and 6 well plates once confluences reached 80%. The final converted cells were put into a 40 ml Ex-Cell CHO medium flask and cultured for another 14-18 days until considerable amounts of antibodies were produced. To settle the dead cells, the cultural supernatant was collected and centrifuged at 1200 rpm for 5 minutes. The supernatant was filtered through 0.45 nm syringe filtrate and stored before being transferred to the Protein A column.

### Purification of full-length antibody

Cell culture supernatant was collected and centrifuged for 5 minutes at 1200 rpm before filtration. The filtered samples were diluted by loading buffer 1:1 and poured into a Protein A- Sepharose resin column. The column was washed with 5 times the column volume of loading buffer (pH 7.2) and eluted with sodium citrate (pH 3.0). The acidic pH of eluted samples was immediately neutralized (pH 7.0) with a 0.5 M Tris buffer (pH 9.0). Aliquots were collected for SDS-PAGE analysis. The antibody was quickly frozen at -80°C.

### ELISA

ELISA and confocal microscopy were used to confirm the bioactivity of purified antibodies and culture supernatant. A 96-well plate was coated with the PD-L1 antigen of interest and incubated at 4°C overnight. The wells were emptied and washed three times with PBS. After washing, the wells were blocked for 2 hours at 37°C with 200 µl 3% BSA in PBS, followed by washing with PBST. The 100 µl supernatant was added and incubated for 2 hours at 37°C. Wells were washed three times with PBST before being loaded with 100 µl rabbit anti-human IgG-HRP antibody and incubated at 37°C for 1 hour. After washing the steps, 100 µl substrate tetra-methyl-benzidine (TMB) was added to develop the color for 30 minutes at 37°C in the dark. The reaction was halted by adding 2M H_2_SO_4_ at 37°C for 10 minutes. Anti-PDL1 antibody binding affinity was evaluated using an ELISA reader with a 450 nm absorbance. All of the analyses were done in triplicate.

### Immunofluorescence analysis

A549, BEL-7402, and MDA453 cancer cell lines were used for immunofluorescence analysis. Cells were seeded at 2x10^4^/ml onto glass coverslips in regular serum-containing RPMI 1640 medium and incubated at 37°C until 80% confluence. Cells were washed three times with ice-cold PBS (5 minutes between washes) and fixed with 4% paraformaldehyde. After three washes, an anti-PDL1 purified antibody from transformed CHO cell extracts was added and incubated at room temperature for two hours. The slides were washed three times before being loaded with a rabbit anti-human IgG FITC conjugated antibody (dilution 1:1000) for 1 hour at 37°C, followed by a 10-minute DAPI incubation. Coverslips were inverted on the slide and viewed through a Zeiss fluorescence microscope with a 63x oil immersion objective (Zeiss, Germany).

### Flow cytometry determination

Flow cytometry was used to detect the antibody’s binding to a surface antigen on A549 cells. In the 1ml FITC buffer (PBS supplemented with 1% FBS), cells were inoculated with anti-PDL1 for 30 minutes on ice. After washing, the cells were incubated for 1 hour at 4°C with FITC labeled rabbit anti-human IgG (H + L) antibody, and the flow binding interaction was examined.

### Molecular modeling

The purpose of homology modeling is to get the lowest energy conformation. Though there is numerous model in complex with other targets embedded in the protein data bank for PD-L1, upon retrieval, we observed that the PD-L1 adopted a different conformation, hence we decided to model the structure. Both, the three-dimensional (3D) structural coordinates were modeled using SWISS-MODEL (28). The amino acid sequence of both PD-L1 and anti-PDL1 scFvs were submitted to the SWISS-MODEL web server to generate the models based on query sequence coverage and identity. The most reliable 3D structure was selected based on the Global Model Quality Estimation (GMQE) (29) and Qualitative Model Energy Analysis (QMEAN) (30) values. The GMQE values are usually between 0 and 1, and the higher the number, the higher the reliability of the predicted structure, while for QMEAN, a value below 4.0 shows reliability (31). Moreover, the protein (PD-L1)–protein (anti-PD-L1) complex analysis was performed using ZDOCK server. PPIs are essential to cellular and immune function, and in many cases, due to the absence of an experimentally determined structure of the complex, these interactions must be modeled to obtain an understanding of their molecular basis. The complexes were visualized using molecular operating environment (MOV) v2022 (*Molecular Operating Environment (MOE)*, 2022.02 Chemical Computing Group ULC, 1010 Sherbooke St. West, Suite # 910, Montreal, QC, Canada, H3A 2R7, **2022**.).

### Statistical analysis

All graphical and numerical analyses were analyzed with GraphPad Prism 5 (GraphPad Software; Inc: La Jolla, CA), Macromedia Fireworks 8, and CorelDraw X7. One-way analysis of variance using Prism 5 software was applied to evaluate the binding affinity of expressed anti-PDL1 antibodies. All tests were performed in triplicate. The significance level was set at p ≤ 0.05.

## Results

### Recombinant plasmid development

**Figure 1:**
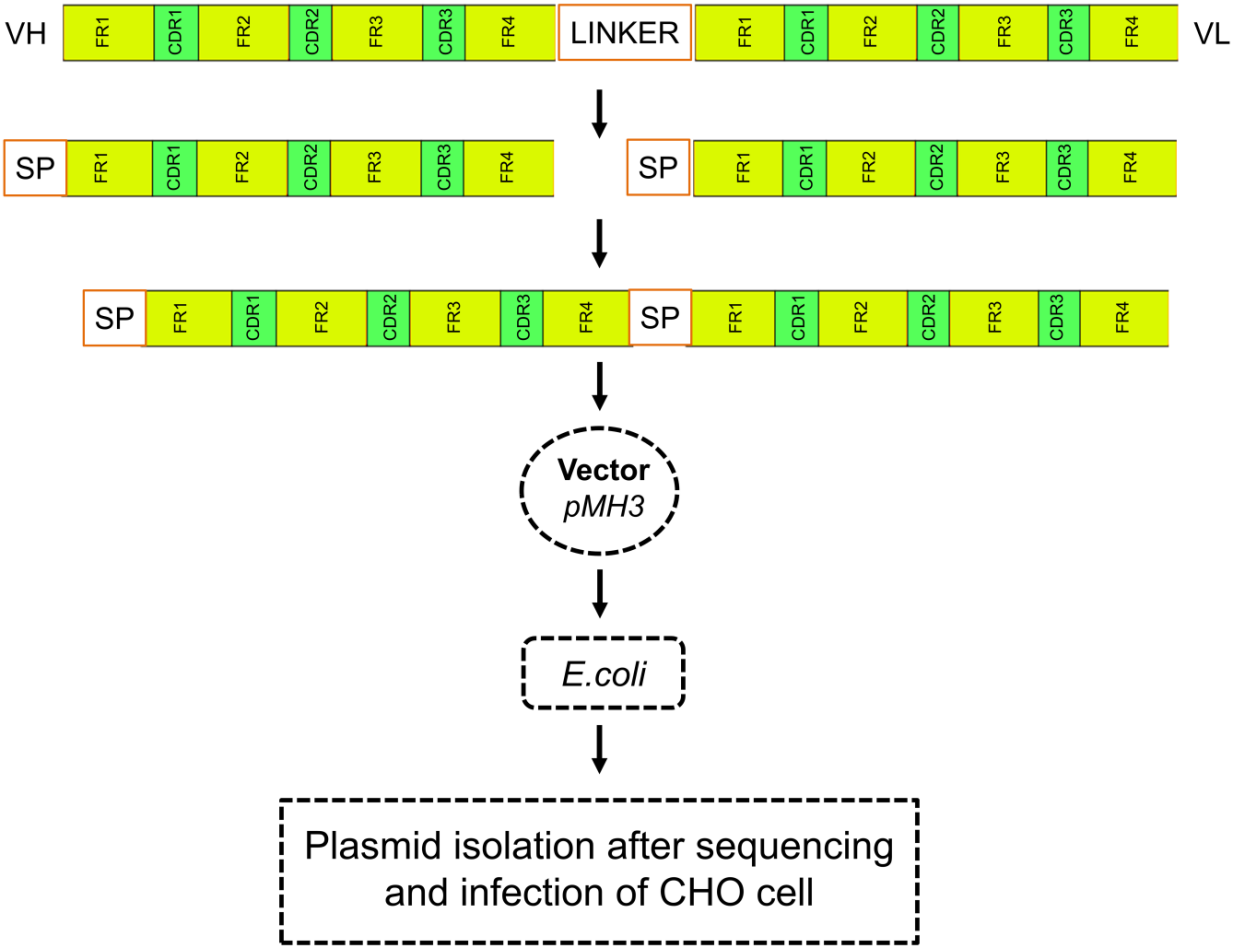
Schematic diagram of recombinant construction. Heavy and light chains, as well as their complementary determining regions (CDR), were polymerized and ligated with signal peptides in an scFv-containing linker intermediate. The recombinant construct was cloned into the pMH3 vector and transfected into bacteria. Positive clones were selected, and plasmids containing the necessary immunoglobulin fragments were collected and used for full-length antibody transfection in CHO cells.

The DNA sequence of anti-PDL1 scFv was used to develop a recombinant full-length antibody. Our research group had previously created the scFv molecules. As illustrated in **Figure 1**, a full-length antibody construct was designed and inserted into a CHO cell for efficient production. The heavy and light chains, as well as their complementarity determining regions (CDR), were polymerized and bound by a linker peptide intermediate. These recombinant constructs were cloned into the pMH3 vector and tested in bacterial cells for recombinant product development success. Positive clones were chosen. Plasmids with suitable immunoglobulin segments were gathered and used to transfect full-length antibodies into CHO cells.

**Figure 2:**
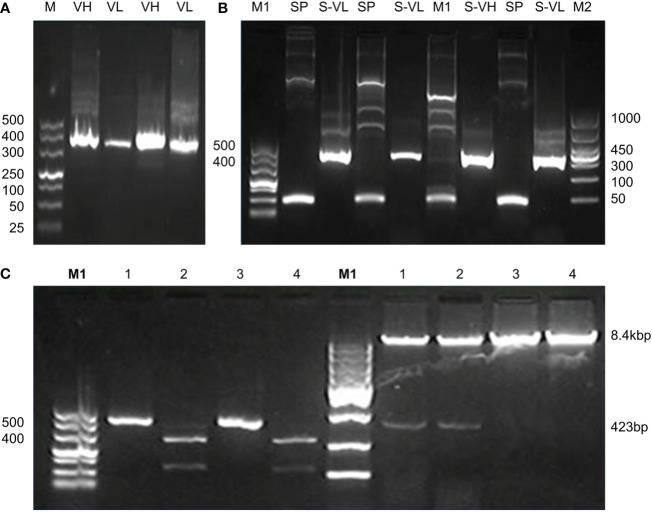
**(A)** Polymerization and ligation of signal peptides with VH/VL fragments. Panel A shows PCR confirmation of VH and VL regions. M represents 500 base pairs of molecular markers, VH and VL indicate variable fragments of the heavy chain and light chain. **(B)** Indicate signal peptide polymerization and its ligation with variable regions. The M1 shows 500 base pairs and the M2 indicates 1000 base pairs of molecular markers. SP indicates 60 base pairs signal peptide, S-VH shows overlapping of signal peptides with the heavy chain of approximately 423 base pairs bands, and S-VL shows overlapping results of a signal peptide with a variable chain of 381 base pairs bands. The tests were repeated twice to configure the integrity. **(C)** Recombinant confirmation of heavy chain variable regions by PCR and enzyme activity. The PCR validation of the heavy chain overlapping construct is shown in the left panel of the figure. Positive findings were seen in Lanes 1 and 3. Restricted enzyme activity is shown in the right panel. A positive construct may be seen in Lanes 1 and 2.

**Figure 3:**
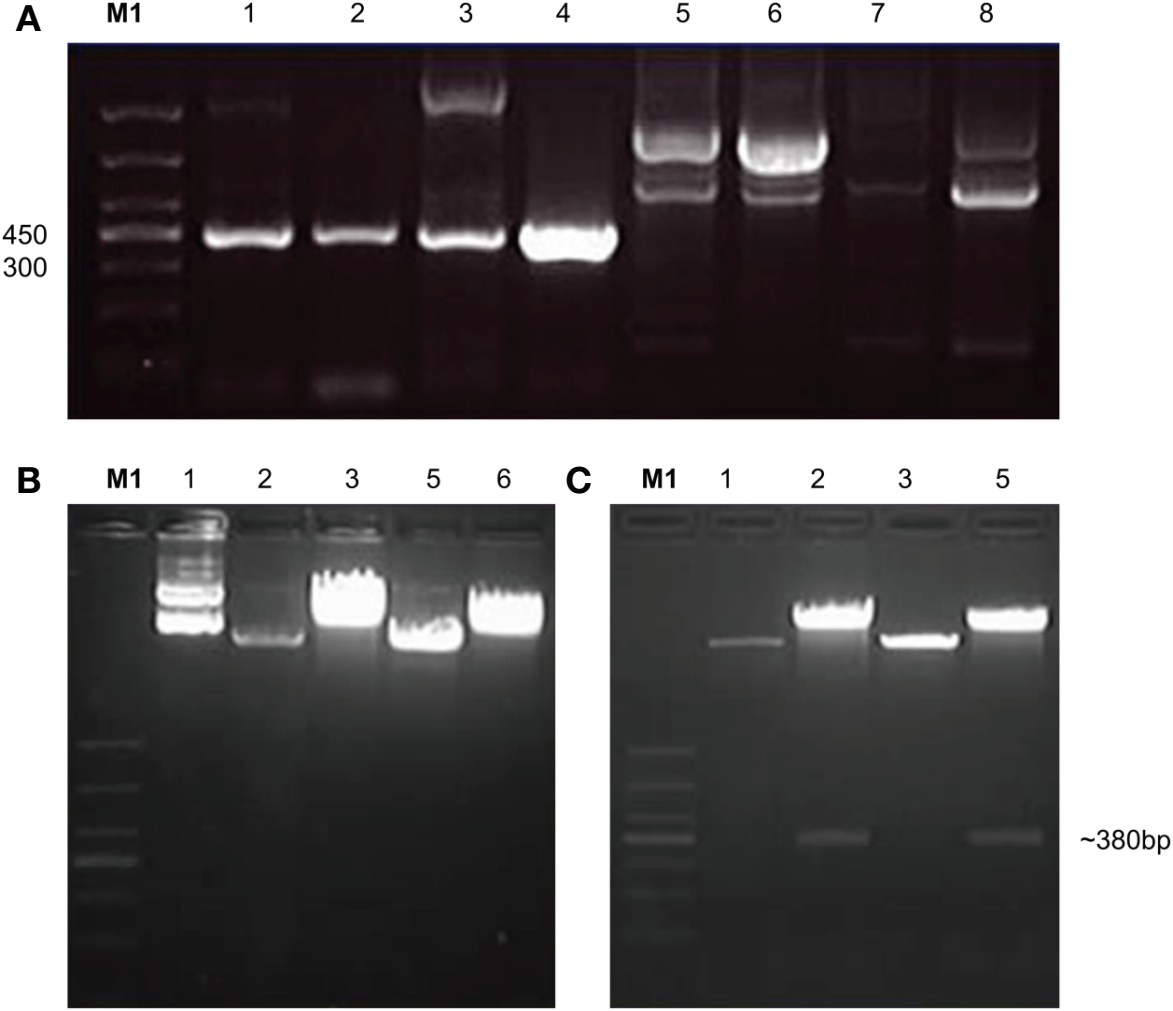
Confirmation of variable regions by PCR and enzyme activity of light chain. **(A)** indicates PCR confirmation of variable recombinant. Lanes 1-4 showed positive results and lanes 5-8 were declared negative. The positive products were further analyzed by double enzyme digestion. **(B)** shows the activity of *EcoRI* enzyme which was recorded as the first enzyme, **(C)** shows the second enzyme *BsiwI* restriction enzyme activity.

**Figure 4:**
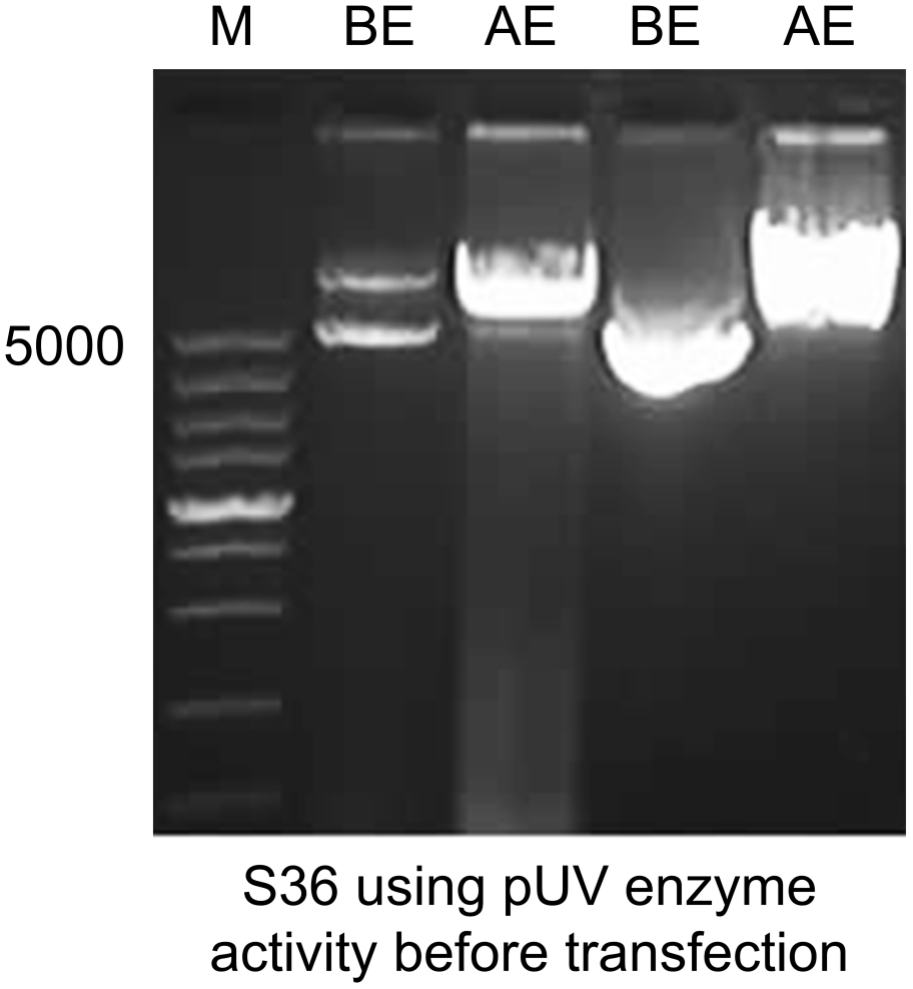
Linearization of the final recombinant before transfection. Immunoglobulin variable construct was treated with pUV restriction enzyme and was loaded on 1% agarose gel. Before enzyme activity, the plasmid showed two band copies. After enzyme activity, a single band high localization was recorded. M represents 5000 base pairs molecular marker. The first two lanes show heavy chain fragments before and after enzyme activity. The last two show before and after enzyme digestion for light chain chimeras.

The signal peptides, VH and VL sequences were polymerized, and the products were loaded on 1% agarose gel as shown in **Figure 2A**. The results showed 60 bases of the signal peptide, 363 base pairs in the VH region, and 321 bases in the VL region. Correct localization of the gel against molecular markers confirmed the predicted band sizes. After confirmation of the desired heavy and variable regions, the signal peptide sequences were ligated with VH/VL sequences *via* overlap PCR, as shown in **Figure 2B**. The polymerization and ligation processes were repeated twice to ensure that targeted sequences were oriented correctly. Restriction enzymes were used to digest extracted purified products. To facilitate the digestion and ligation, we added *Eco*RI and *Nhe*I enzyme sites to signal peptide and heavy variable regions. *Eco*RI and *Bsiw*I were also added to variable fragments in the same way. Because these enzyme sites were also present in the pMH3-VH/VL DNA sequences, ligation was effective. The overlapped digested fragments were ligated to pMH3kH and pMH3kL plasmid. The final ligated products were transfected into *E. coli* DH5 and positive clones were selected for sequencing. The recombinant construct was also validated using PCR and restricted enzymes, as illustrated in **Figures 2**, **3**. **Figure 4** shows how the recombinant constructs were linearized before transfection using the pUV enzyme. Before the enzyme digestion with pUV, two bands appeared on the gel, whereas after digestion, only one extended band was formed. Single bands were purified using a DNA digestion kit, and VH and VL concentrations were determined.

### Mammalian cell transfection

**Figure 5:**
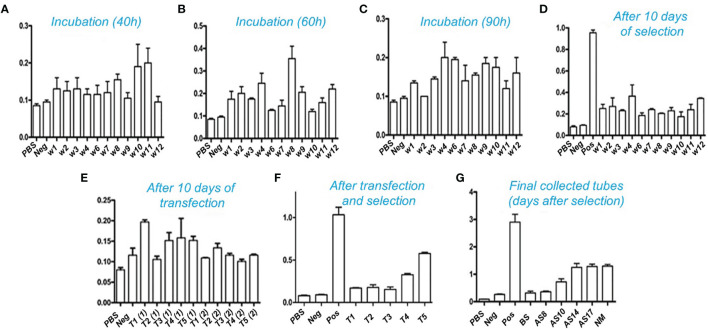
Affinity determination with ELISA: Binding interaction of the antibody with PD-L1 antigen after transfection and selection. Positive transformed cells were selected and remained incubated for up to 10-14 days. Cultural supernatants were taken from five different wells. The transfection processes were repeated twice in triplicate for each. High titer clones were further selected for 96 wells plates to isolate the single positive clones. **(A–C)** show the incubation time of a single clone selection. A total of 12 clones were selected after 96 wells plates single cell inoculation. These clones were further utilized for expression and purification. **(D)** shows an intermediate collection after 10 days of selection and **(E)** indicates collection after 10 days of transfection. The high-affinity titer supernatant was selected and tested for binding through ELISA as shown in **(F)**. **(G)**: indicates the overall results of collected supernatant up to 17 days of incubation transformed CHO cells into serum-free media. BS: Before selection, AS: After selection, Pos: Positive, NM: indicates results after purification through the Protein A resin column.

The mammalian CHO cells were cultured in DMEM medium supplemented with 10% FBS for up to two generations. Initially, all transfections were carried out on 6-well plates. To allow transfection, the linear recombinant pMH3-kH/kL vectors were incubated with Lipofectamine 6000. Following incubation, the cells were allowed to grow in a selective medium containing 700µg/ml G418 to isolate transformed cells. The supernatant was collected for a period of up to ten days. ELISA was used to measure binding affinities. The transformed cells were then seeded into 96-well plates to create a single-clone library. The cells were maintained for up to 10-14 days. The supernatants were collected regularly and analyzed using ELISA to determine the expression of the anti-PDL1 fusion protein. As illustrated in **Figures 5A–E**, the transformed single clone of 12 separate positive clones from a 96-well plate was further processed for efficient binding affinity tests. The ELISA results of a single highly positive clone T5 were quarantined and kept on serum-free ex-cell media for further study as shown in **Figure 5F**. Cells were kept alive for 14 to 18 days. The expressed supernatant showed homogeneous interaction after 10 days of incubation, as illustrated in **Figure 5G**, and was purified further using a Protein A resin column. NM in **Figure 5G** represents the binding affinity of the purified products obtained by using Protein A resin.

### Full-length antibody purification

In supernatant media, recombinant CHO cells secrete anti-scFv of PDL1 chimera. The cultural supernatant was collected and centrifuged at a low speed. Protein A resin column was used to purify the antibody. When grown in a 40 mL flask, the final eluted products were analyzed using a standard BCA kit, and the yield was determined, yielding around 0.5-0.8 mg/mL pure products. The purified products were subjected to SDS-PAGE analysis and monomeric fragments and di fragments as indicated in the (+) sign. Antibodies were loaded on SDS-PAGE and counterstained with a silver reagent as shown in **Figure S1**. Many bands containing the required immunoglobulin emerged under non-reducing conditions.

### Bioactivity determination

**Figure 6:**
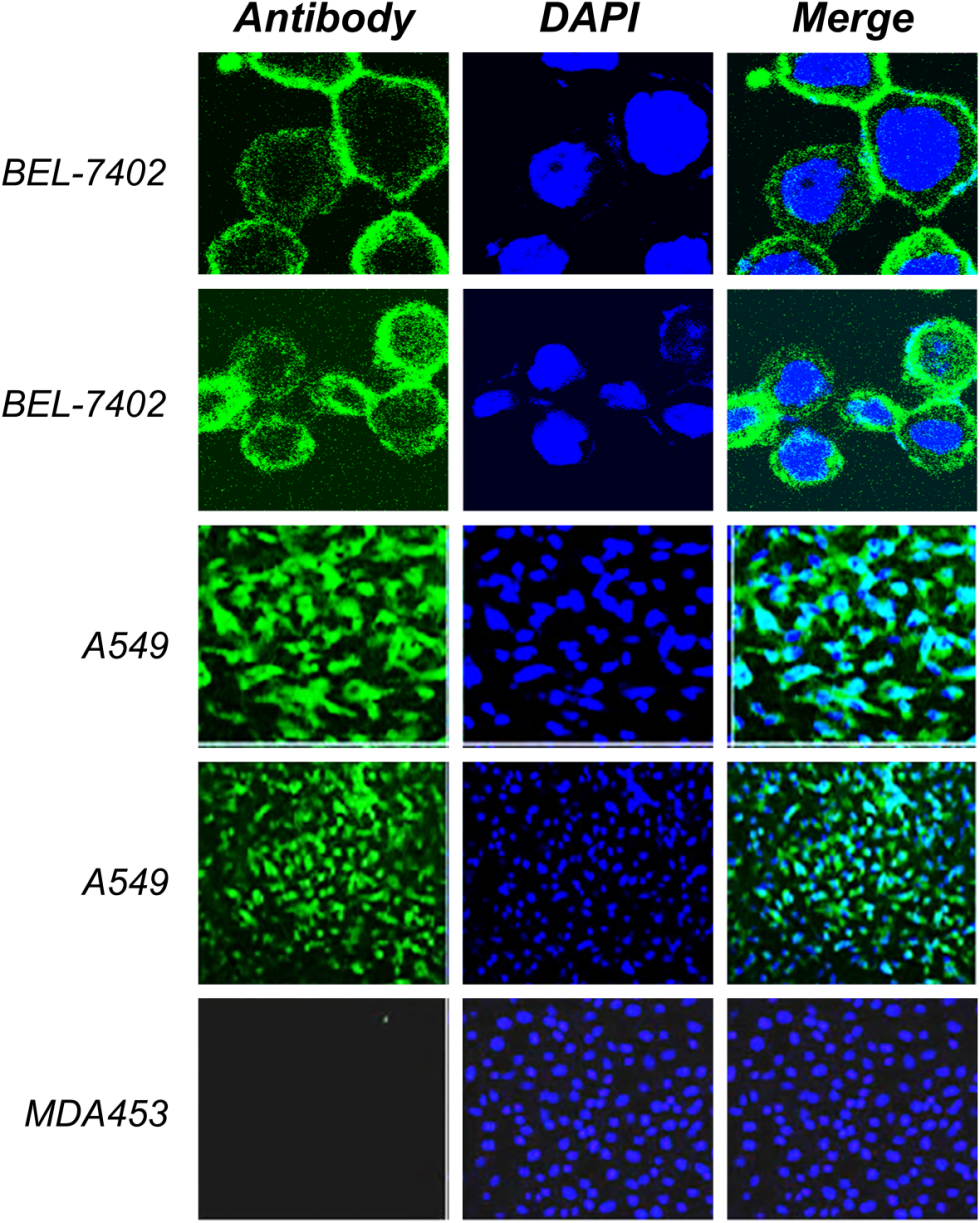
Immunofluorescence analysis. The positive cell lines (A549, BEL-7402) and negative MDA453 cells were cultured and fixed on the cover glass. The cultural supernatant was used as the primary antibody and rabbit anti-human FITC conjugated antibody was used as the secondary antibody. Results indicated a high binding affinity with positive cells and no such binding was observed in negative cells. DAPI was used for visualization of the nucleus.

**Figure 7:**
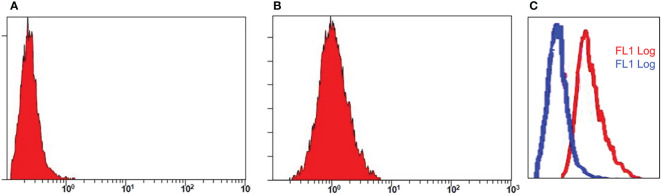
Flow cytometry analysis. Assay represents fluorescence intensity of PD-L1 antibody in negative cell lines, MDA 453 **(A)** and positive cell lines, A549 **(B)**. The histogram shifts towards right in **(C)** indicates high intensity and binding affinity of anti-PD-L1 antibody for A549 cells.

Using ELISA and immunofluorescence, the purified products were further tested for bioactivity. PD-L1 antigen was coated in 96-well plates, and purified antibody supernatant was allowed to attach to the antigen. To test recombinant PD-L1 antibody binding affinity, HRP conjugated rabbit anti-human-IgG was added. The results demonstrated that the purified chimera was correctly oriented and bioactive. The membrane-binding affinity of a monoclonal antibody derived from recombinant CHO cell supernatant was evaluated against the cancer cell lines A549, BEL-7402 and MDA453. As shown in **Figure 6** of immunofluorescence analysis and **Figure 7** of flow cytometry detection, fluorescence signaling was discovered on positive cell lines (A549, BEL-7402) while no such signals were detected on negative MDA453 cells. In PD-L1 positive cancer cells, the purified antibody has a high surface binding affinity and can thus be used for therapeutic enrichment.

### Molecular modeling

**Figure 8:**
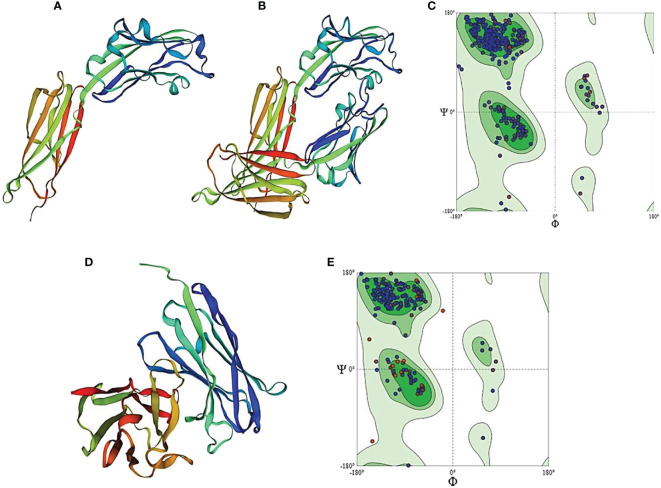
Homology model of PD-L1 antigen and antibody generated by Swiss-Model Homology Modeling. **(A)** represents PD-L1 monomer, **(B)** indicates homo-dimer of PD-L1, and **(C)** shows Ramachandran plot for PD-L1 protein. **(D)** shows homology model of anti-PDL1 scFv generated by SWISS model. **(E)** represents Ramachandran plot for scFv-PDL1 protein.

The Protein Data Bank (PDB) is the primary database on 3D biological molecule structures. The 3D structure of PD-L1 was retrieved from the data bank by using amino acids of the extracellular domain to generate the 3D model of PD-L1 protein as shown in **Figures 8A, B**. The sequence similarity coverage was found 100% under the template ID 4Z18 named programmed cell death 1 ligand 1. The GMQE value was found as 0.89 and the QMEAN was obtained as 0.83 showing an acceptable range as per the experiment value. The Ramachandran plot of PD-L1 antigen showed about 96.88% residues in the favored region, 2.88% residues in the allowed region, and 0.24% residues in the outlier region. Thus, overall 99.76% of residues were found to be in the allowed region as shown in **Figure 8C**.

As the 3D structure of scFv-PDL1 is not available in the protein data bank, we used homologous modeling to generate the 3D model as shown in **Figure 8D**. The PDB data bank was checked for the 3D structure of the selected scFv-PDL1 and it was confirmed that no 3D structure had been crystalized or modeled to date. We used SWISS modeling to predict the structure. The sequence maximum similarity between targets and templates was found to be 64% (ID: 5F72), 68% (ID: 5WN9), and 77.37% (ID: 6EHY) respectively. The Ramachandran plot of scFv-PDL1 showed about 95.56% residues in the favored region. The 4.04% residues were in the allowed region and 0.40% residues in the outlier region. Overall 99.6% of residues were found to be in the allowed region. The GMQE value was found 0.79 and the QMEAN score was 0.78 showing an acceptable range as shown in **Figure 8E**.

### Molecular docking and visualization

**Figure 9:**
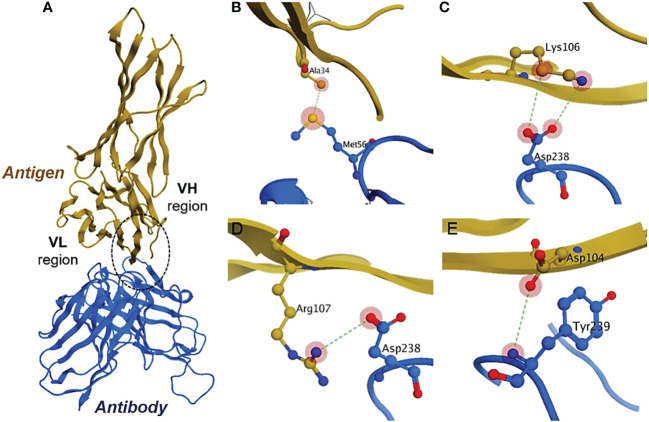
Antigen-antibody interaction analysis. **(A)** show the binding interactions of PD-L1 and scFv molecule. The highlighted encircle shows the CDRs interactions with antigen sites. **(B–E)** models indicate the site-specific hydrogen interactions between amino acid residues.

PPI simulations reveal hydrogen and ionic bonds. The docked complexes from the ZDOCK server were further analyzed by MOE v2022. We have selected the top-1 complex based on the interaction profile between the antigen (PD-L1) and antibody (scFv-PDL1). The results indicate that mostly the residues belonging to the VH region interacted with the antigen more than the VL region. The detail for antigen and antibody interaction has been enlisted in **Table 2** and the graphical representations of interactions are shown in **Figure 9**. The figure highlights the conserved interactions of a given site when interacting with a targeted partner. The yellow protein is the antigen interacting with an antibody. The binding residues are shown in red, blue, and yellow in panels **B, C, D**, and **E** belonging to the PD-L1 and anti-PDL1. They are utilized to form H-bonds with both partners,

**Table 2:** The detail of homology modeling and protein contact analysis.

Homology Modeling			
Model	Template/identity (%)	GMQE	QMEAN
Antigen	4Z18/100	0.89	0.83
Antibody	5WN9/68	0.79	0.78
**Protein-Protein Contacts Analysis**			
**Antibody Residues**	**Antigen Residues**	**Energy (kc/mol)**	**Distance (Å)**
Met56	Ala34	-0.600	3.268
Asp238	Lys106	-11.500	3.565
Asp238	Arg107	-1.800	3.494
Tyr239	Asp104	-0.500	3.748

**Figure 10:**
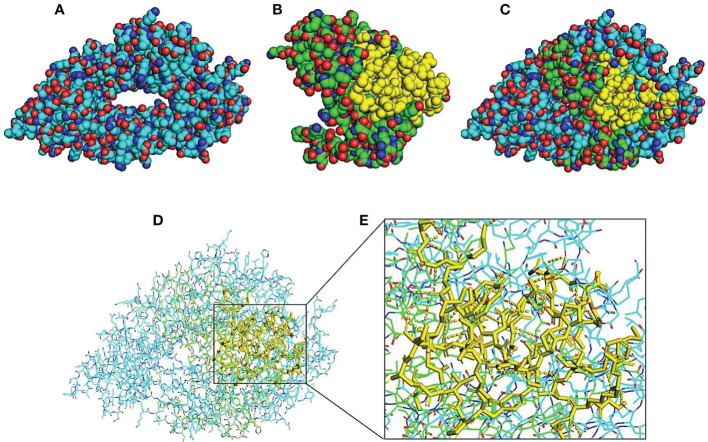
Molecular visualization and interaction 3D structure analysis and visualization by using PyMOL software integrated with Python. Models **(A, B)** indicate 3D structures of PD-L1 and scFv-PDL1. The yellow regions indicate the CDR regions of the scFv molecule. Model **(C)** indicates a merged model of both antigen and antibody fragment and **(D)** shows surface binding interaction. H-bonding interactions are indicated in dotted lines in **(E)**.

The 3D modeled structures of both PD-L1 and scFv-PDL1 were fetched from SWISS model integrated PDB IDs as shown in **Figures 10A, B**). The basic docked models of both antigen and antibody fragments were shown in **Figure 10C**. The surface binding was evaluated as shown in **Figure 10D**. Moreover, the results were double-checked using Chimera which is graphical presentation software for proteins and tiny molecules (32). Other than the MOE v2022, we also utilized the Chimera to evaluate the H-bonding between the targeted antigen and antibody fragment as enlisted in **Tables S1, S2** and **Figure 10E**. A range of 73 to 86 interactions were discovered among all tested samples, demonstrating that the PD-L1 and its binding antibody molecule had a high binding affinity.

## Discussion

The PD-L1/PD-1 pathway is a well-known immune checkpoint mechanism that is considered a viable target in cancer immunotherapy. Indeed, PD-L1 expression has been detected in a variety of solid tumors, and over-activation of the PD-L1/PD-1 pathway results in a low patient survival rate. Breaking the PD-L1/PD-1 interaction by blocking either cancer or the immunological side of the axis is now employed as an anti-cancer therapy to re-establish a tumor-specific immune response. Wide ranges of blocking antibodies are now available for this purpose. To date, the FDA has approved three anti-PD-L1 antibodies: atezolizumab, durvalumab, and avelumab (33)

Cloning and gene expression in diverse expression systems have both been revolutionized by recombinant approaches. For fast antibody development and expression, a variety of suitable techniques are being used. One of the successful approaches for producing highly specific antibody fragments is optimizing phage display technology for scFv synthesis. Traditional polyclonal antibody manufacturing can be replaced with recombinant antibody synthesis. This method can be used to generate antibodies against tumor surface antigens and their ligands (34). We previously reported on the successful optimization of the extracellular domain of programmed cell death ligands-1, which was then processed to produce single chain variable fragments, scFv-PDL1 (35, 36). The most common host cell machinery used in the manufacture of therapeutic monoclonal antibodies is CHO cells. Aside from the great potency and benefits of using CHO cells in biopharmaceuticals, its development is hampered by the fact that it takes a long time and costs a lot of money (37). Many studies have been published that aim to increase the potency of CHO cell generation while working with restricted resources (37–40).

Using the scFv-PDL1 DNA sequences, we were able to achieve high-affinity full-length PD-L1 antibody expression. The goal of the experiment was to polymerize both VH and VL segments from previously recovered scFv-PDL1 extracts and ligates them with IgK sequence peptides. Through overlapping PCR, the conjugate was ligated to the pMH3 light and heavy chain plasmids. The final pMH3-VH/VL products were introduced to bacterial cells and sequencing analysis validated them. The plasmid was linearized with the pUV enzyme and then transfected to CHO cells using Lipofectamine 2000 (Invitrogen). G418 was used to screen the transfected cells, and a single cell with high expression was chosen. Antibody affinity was measured by ELISA using the medium supernatant collected at regular intervals. The bioactivity of the expressed antibody was validated in A549 and BEL-7402 PD-L1 positive cell lines. Acrcio et al., found novel small chemicals by using in silico analyses paired with *in vitro*, ex vivo, and *in vivo* experimental experiments, that can modify PD-1/PD-L1 interaction and increase T-cell function. They discovered a novel promising group of small-molecule candidates that modulate the PD-L1/PD-1 signaling pathway, allowing effector CD8 T cells to infiltrate the tumor microenvironment in large numbers (41).

Drees et al., reported that single-chain fragments from phage display technology were used to immunize chickens against murine PD-L1. They went on to analyze the expression of variable areas in the bacterial host machinery and found that it might be used for full-length development (42). Antibody generation against PD-L1 was largely done using hybridoma techniques, and no investigations involving CHO host machinery have been published. Despite advancements in recombinant technology, we wish to highlight the sensitivity of antibody synthesis using CHO cell host cell machinery. Using recombinant full-length antibody synthesis, transfection, and cultural techniques, we discovered that CHO cell transfection can help achieve a high yield of PD-L1 antibodies.

Immune checkpoint molecules are intriguing anticancer targets, with therapeutic antibodies targeting the PD-1/PD-L1 pathway being widely used in clinical practice and showing significant promise. However, due to low response rates in specific malignancies, a lack of established biomarkers, immune-related toxicity, innate and acquired drug resistance, and other factors, this treatment is severely constrained. Overcoming these restrictions will greatly broaden the anticancer uses of PD-1/PD-L1 inhibition and enhance cancer patients’ response rate and survival time (43).

An important aspect of an antibody is the epitope that it can detect. The antigenic epitopes that these antibodies target differ, even though they disrupt interactions between PD-1 and PD-L1 (or PD-L2) (44, 45). Similar studies were conducted by Ghaderi et al., where they used panning techniques to isolate several scFvs against the extracellular domain of the PD-1 protein from a human semi-synthetic phage library. After panning, a novel anti-PD-1 scFv (SS107) was discovered that binds to PD-1 antigen and stimulates Jurkat T cells. The chosen anti-PD-1 scFv could restore IL-2 and IFN- production by Jurkat T cells co-cultured with PD-L1 positive tumor cells (46).

However, naked mAbs typically have fewer severe adverse effects when compared to chemotherapeutic medications. Still, some people may experience problems as a consequence. A protein called VEGF that influences the development of tumor blood vessels is the target of the monoclonal antibody bevacizumab (Avastin). It may have adverse effects including elevated blood pressure, bleeding, inadequate wound healing, blood clots, and kidney damage. An antibody known as cetuximab (Erbitux) targets the EGFR cell protein, which is present in healthy skin cells (as well as some types of cancer cells). Some people who use this medication may get severe rashes (47, 48). The production process, avidity, effector role, and delivery to the target tissue are factors to be taken into account when deciding which types of monoclonal antibodies to produce (e.g. a smaller scFv may be able to penetrate a tumor more effectively than a full-sized antibody). Pharmaceutical companies continue to show a strong interest in developing monoclonal antibodies despite their limitations for therapeutic and diagnostic usage. From a clinical and financial standpoint, this will undoubtedly determine the future of treatment and management of common, chronic illnesses (49).

Studies focused on identifying interactions between mAbs and their target antigens may improve mAb binding specificity and efficiency. Wang et al., identified MW11-h317, a high-affinity antibody that specifically binds human PD-1 and effectively blocks PD-1/PD-L1 and PD-1/PD-L2 interactions, using hybridoma screening and antibody humanization. They also discovered that MW11-h317 has an antitumor N-linked glycosylation antigen-binding site. The crystal structure of the PD-1/MW11-h317 Fab complex shows that PD-1 loops and glycosylation are both involved in recognition and binding, with Asn58 glycosylation playing a critical role (50).

Maute et al., explored directed evolution using the yeast-surface display to create the PD-1 ectodomain as a high-affinity (110 pM), a competitive antagonist of PD-L1 to ascertain if PD-1: PD-L1-directed immunotherapy may be boosted with smaller, non-antibody therapies. High-affinity PD-1 revealed better tumor penetration than anti-PD-L1 monoclonal antibodies without causing the depletion of peripheral effector T cells. In syngeneic CT26 tumor models, high-affinity PD-1 was successful in treating both small (50 mm3) and large tumors (150 mm3), whereas the action of the anti-PD-L1 antibody was eliminated against large tumors, which is consistent with these benefits. In addition, they discovered that high-affinity PD-1 could be radiolabeled and used as a PET imaging tracer to effectively differentiate between PD-L1-positive and PD-L1-negative tumors in living mice, offering an alternative to invasive biopsy and histological examination. These findings consequently emphasize the advantageous pharmacology of tiny, non-antibody therapies for improved immunological diagnostics and cancer immunotherapy (51).

The 3D structures of PD-L1 and scFv-PDL1 provide significant information on the function of the proteins’ molecular bases (52). Therefore, high-resolution 3D models of proteins are a key aspect of understanding molecular and biochemical activities (53). The identification of protein structures in an experimental setting proved challenging, time-consuming, and expensive. As a result, homology modeling becomes a useful tool for closing the gap between known protein sequences and experimentally determined protein structure. The SWISS model generates an automated protein structure by selecting templates and aligning target-template sequences (28). Homology modeling is an accurate and precise computational prediction technique for 3D structures (54). The probable residues that serve a vital function in PPI might be discovered using the primary residue prediction method. (55). The PPI has been discovered on a huge scale in the past using laboratory techniques, but these approaches were difficult to apply to new target organisms. As a result, docking studies are utilized to anticipate interactions between PD-L1 and scFv-PDL1, which are connected to form extremely stable complex structures with low energies and a better possibility of contact. Docking and computational approaches are thus required for antigen-antibody interaction and binding affinity determination (56, 57).

In comparison to laboratory methods, our findings indicated that computational techniques, i.e., PP-docking could accurately anticipate interactions between PD-L1 and anti-PDL1 molecules and that they were cheaper, easier, and more effective. The anticipated results showed that the antigen-antibody interaction prediction had structural support for reciprocal recognition, as well as interactions between PD-L1 and its antibody fragment. It is critical to comprehend the relationship between PD-L1 and scFv-PDL1. The CDRs of scFv-PDL1 fragments frequently have docking sites in the VH region that can interact with the PD-L1 antigen (58).

Cancer continues to be one of the primary causes of death. Immunotherapeutics have steadily become a hot spot for tumor treatment in recent years, as our understanding of tumor immunotherapy has improved. Among these, PD-1/PD-L1 inhibitors such as nivolumab and pembrolizumab, as well as atezolizumab, avelumab, and durvalumab, be highly effective in a variety of tumor types. It has been established that these inhibitors play a vital role in the anti-tumor process, considerably enhancing patient survival and delaying the progression of the underlying malignancy. However, their manners of therapeutic interference and potential for immune system damage have raised concerns about their applicability (59). Blocking the PD-L1/PD-1 pathway is highly effective cancer immunotherapy. The pathway-targeting therapeutic monoclonal antibodies (mAbs) approved by the FDA have a strong affinity, blocking capability, and low antibody effector activity. Several rat antimouse mAbs have been employed in mouse models of cancer immunotherapy (60).

PP-docking is the only computational method for simulating physical interactions between proteins. (61, 62). However, as in several previous research (63, 64) it is vital to compare the results of PP computation with experimental data. The flexible docking techniques are limited by their computational complexity, and they are only seldom used in real protein docking now. Sierra S. Beach, et al., developed a method that combines molecular dynamics simulations with Fold X to predict free energy changes in F protein folding and binding to the motavizumab antibody upon each conceivable amino acid substitution. They investigated eight predicted escape mutations in an infectious clone. Replication-effective viruses were seen for each selected mutation, which was consistent with our assumptions about F protein stability (65). Computational research revealed that PD-L1 binds more strongly with scFv antibody fragments. We may conclude from this investigation that the generated antibody has a high affinity for PD-L1. We performed wet-lab studies using phage display techniques, ELISA, flow cytometry, and immunofluorescence analysis to assess the efficacy and accuracy of the affinity approach. It will make it easier to design biological studies to investigate the interaction impact. The existing laboratory and computational data could be useful in the development of novel antibody fragments to enrich bio-therapeutics in cancer therapy.

## Conclusion

Alternatives to traditional polyclonal antibody synthesis include recombinant antibody production. Antibody gene fragments can now be genetically modified, cloned, and expressed to preserve higher binding affinity and improved pharmaceutics. Because the heavy and light chains of immunoglobulin are transfected separately, developing an expression system in mammalian cells is slow and difficult. We’ve created a new PD-L1 antibody with a high affinity and bioactive novelty. These approaches can be used to express full-length antibodies, according to our findings. The capacity of the full-length antibody shown here to detect the target antigen will aid in the development of new clinical investigations into tumor immunology and immune responses.

Protein-protein interactions maintain a vast range of biological activities, including cell division and metabolic processes. The detection of PPI using laboratory procedures is expensive, time-consuming, complicated, and demanding. However, for the prediction of PPIs, computational algorithms were employed. The same PPI mechanism was utilized to find antigen-antibody interactions using a docking approach. The Swiss model was utilized for homology modeling, while MOE v2022 and Chimera were used to validate the 3D models. The Ramachandran plots indicated a value of over 90% for high-quality structures. Our findings show that both wet-lab and in-silico studies are crucial in identifying and docking PD-L1 antigen and antibody affinity. Antigen-antibody interactions can be accurately determined with this investigation. The present methodologies of both wet-lab and computational studies of evaluation can be productive in novel pharmaceutics development in a variety of cancers by blocking PD-L1/PD-1 interactions.

## Data availability statement

The original contributions presented in the study are included in the article/**Supplementary Material**. Further inquiries can be directed to the corresponding authors.

## Author contributions

All the mentioned authors have made a significant, direct, and intellectual contribution to the work, and have granted approval to be published.

## Funding

This work was supported by the National Natural Science Foundation of China (Grant No. 81872784 and 81430081)

## Conflict of interest

The authors declare that the research was conducted in the absence of any commercial or financial relationships that could be construed as a potential conflict of interest.

## Publisher’s note

All claims expressed in this article are solely those of the authors and do not necessarily represent those of their affiliated organizations, or those of the publisher, the editors and the reviewers. Any product that may be evaluated in this article, or claim that may be made by its manufacturer, is not guaranteed or endorsed by the publisher.
