# Supplementary material for: Bioengineering and computational analysis of programmed cell death ligand-1 monoclonal antibody
Source: Front Immunol. 2022 Oct 21;13:1012499. doi: 10.3389/fimmu.2022.1012499 (PMC9633666; doi:10.3389/fimmu.2022.1012499)

Bioengineering and computational analysis of programmed cell death ligand-1 monoclonal antibody

Running title: Bioengineering of PD-L1 monoclonal antibody

Muhammad Kalim[1,5*], Hamid Ali[2*], Ashfaq Ur Rehman[3], Yong Lu[4,5] and Jinbiao Zhan[1*]

[1]Department of Biochemistry and Cancer Institute of the Second Affiliated Hospital, Zhejiang University, School of Medicine, Hangzhou, China
[2]Department of Biosciences, COMSATS University, Islamabad, Pakistan
[3]Department of Molecular Biology and Biochemistry, University of California, Irvine, CA, United States
[4]Laboratory of Minigene Pharmacy, School of Life Science and Technology, China Pharmaceutical University, Tongjia Xiang, Nanjing, P.R. China
[5]Current Address: Houston Methodist Cancer Center/Weill Cornell Medicine, Houston, TX, United States

Correspondence should be addressed to:

*Dr. Muhammad Kalim

Email (mkalim@houstonmethodist.org)

*Dr. Jinbiao Zhan

Email (jzhan2k@zju.edu.cn)

*Dr. Hamid Ali

Email (hamidpcmd@yahoo.com)

Table S1: List of H-bonds between PD-L1 and scFv-PDL1 analyzed by Chimera software

(Constraints relaxed by 0.4 angstroms and 20 degrees)

PD-L1	AA Homologue	scFv-PDL1	AA Homologue	Distance (A)
**TYR**	10. A-OH	SER	23.A OG	3.075
**VAL**	11.A N	GLN	8.A OE1	3.148
**THR**	19.A OG1	VAL	VAL	3.249
**ILE**	20.A N	MET	83.A O	2.385
**CYS**	22.A SG	TYR	82.A OH	3.399
**ARG**	68.A NH1	HIS	86.A ND1	3.033
**ARG**	68.A NH2	LEU	85.A O	3.262
**LYS**	71.A NZ	HIS	86.A NE2	3.305
**ASN**	78.A ND2	GLU	12.A OE2	1.747
**THR**	84.A OG1	PHE	66.A O	3.521
**CYS**	96.A SG	TYR	82.A OH	2.943
**THR**	109.A OG1	THR	79.A O	1.790
**LYS**	111.A NZ	ALA	35.A O	3.132
**LYS**	118. A NZ	LEU	47.A O	2.715
**GLN**	121.A NE2	GLN	151.A O	2.175
**THR**	136.A OG1	GLU	215.A OE2	1.816
**CYS**	137.A SG	SER	152.A O	3.242
**GLN**	138.A N	THR	150.A O	2.899
**LYS**	144.A NZ	SER	176.A OG	3.069
**TRP**	149.A N	THR	219.A OG1	3.142
**LEU**	157.A N	THR	165.A OG1	2.351
**THR**	162.A N	GLU	215.A O	2.579
**THR**	163.A OG1	GLY	211.A O	2.489
**THR**	164.A OG1	SER	173.A OG	2.877
**THR**	164.A OG1	GLY	213.A O	3.280
**LYS**	167.A NZ	SER	173.A O	3.468
**ASN**	174.A N	VAL	174.A O	2.533
**SER**	177.A N	CYS	168.A O	2.913
**CYS**	191.A SG	SER	152.A O	2.112
**CYS**	191.A SG	THR	167.A O	1.164
**ARG**	195.A NH1	PHE	110.A O	3.323
**ARG**	195.A NH2	GLN	234.A OE1	2.234
**TYR**	10.A OH	SER	9.A N	2.488
**TYR**	10.A OH	SER	9.A OG	1.561
**ASP**	72.A OD1	SER	18.A OG	2.464
**ASP**	72.A OD2	SER	19.A N	3.105
**LEU**	81.A O	VAL	22.A N	2.898
**LYS**	7.A O	SER	23.A OG	3.271
**TYR**	10.A OH	SER	23.A OG	3.075
**GLU**	21.A O	SER	23.A OG	2.527
**THR**	109.A O	CYS	24.A SG	2.527
**LYS**	7.A O	LYS	25.A N	2.894

**LYS**	87.A O	SER	37.A OG	3.137
**GLU**	13.A O	MET	50.A N	2.559
**GLU**	42.A O	SER	58.A OG	2.300
**GLU**	42.A OE1	SER	58.A OG	3.242
**GLN**	82.A O	THR	71.A N	3.057
**TYR**	63.A O	THR	71.A OG1	3.534
**ASP**	8.A OD1	THR	80.A OG1	3.345
**LYS**	106.A O	TYR	82.A OH	2.814
**THR**	109.A O	CYS	98.A SG	4.030
**LYS**	111.A O	ALA	99.A N	1.638
**CYS**	191.A O	GLN	151.A NE2	3.485
**GLU**	205.A O	SER	154.A OG	2.979
**GLU**	205.A OE2	THR	155.A OG1	1.670
**ASP**	153.A O	SER	159.A N	2.935
**LEU**	157.A O	THR	165.A OG1	3.012
**ILE**	148.A O	ILE	166.A N	1.322
**VAL**	175.A O	CYS	168.A SG	3.785
**THR**	164.A OG1	SER	173.A OG	2.877
**THR**	163.A O	SER	175.A OG	2.984
**THR**	164.A O	TRP	177.A N	2.518
**ALA**	114.A O	TYR	181.A OH	3.337
**GLU**	199.A OE2	LYS	190.A NZ	3.302
**THR**	163.A O	SER	212.A OG	2.642
**CYS**	137.A O	PHE	216.A N	3.214
**THR**	161.A OG1	THR	217.A N	2.750
**VAL**	147.A O	THR	217.A OG1	2.126
**THR**	161.A OG1	THR	217.A OG1	3.099
**TYR**	116.A O	CYS	233.A SG	2.986
**PHE**	173.A O	TYR	236.A N	2.665
**GLU**	140.A OE1	THR	242.A OG1	2.194
**CYS**	191.A O	THR	247.A OG1	2.601

Table S2: List of H-bonds between PD-L1 and scFv-PDL1 analyzed by Chimera software (Constraints relaxed by 0.4 angstroms and 20 degrees)

PD-L1	AA Homologue	scFv-PDL1	AA Homologue	Distance (A)
**TYR**	10. A-OH	SER	23.A OG	3.075
**VAL**	11.A N	GLN	8.A OE1	3.148
**THR**	19.A OG1	VAL	VAL	3.249
**ILE**	20.A N	MET	83.A O	2.385
**CYS**	22.A SG	TYR	82.A OH	3.399
**ARG**	68.A NH1	HIS	86.A ND1	3.033
**ARG**	68.A NH2	LEU	85.A O	3.262
**LYS**	71.A NZ	HIS	86.A NE2	3.305
**ASN**	78.A ND2	GLU	12.A OE2	1.747
**THR**	84.A OG1	PHE	66.A O	3.521
**CYS**	96.A SG	TYR	82.A OH	2.943
**THR**	109.A OG1	THR	79.A O	1.790
**LYS**	111.A NZ	ALA	35.A O	3.132
**LYS**	118. A NZ	LEU	47.A O	2.715
**GLN**	121.A NE2	GLN	151.A O	2.175
**THR**	136.A OG1	GLU	215.A OE2	1.816
**CYS**	137.A SG	SER	152.A O	3.242
**GLN**	138.A N	THR	150.A O	2.899
**LYS**	144.A NZ	SER	176.A OG	3.069
**TRP**	149.A N	THR	219.A OG1	3.142
**LEU**	157.A N	THR	165.A OG1	2.351
**THR**	162.A N	GLU	215.A O	2.579
**THR**	163.A OG1	GLY	211.A O	2.489
**THR**	164.A OG1	SER	173.A OG	2.877
**THR**	164.A OG1	GLY	213.A O	3.280
**LYS**	167.A NZ	SER	173.A O	3.468
**ASN**	174.A N	VAL	174.A O	2.533
**SER**	177.A N	CYS	168.A O	2.913
**CYS**	191.A SG	SER	152.A O	2.112
**CYS**	191.A SG	THR	167.A O	1.164
**ARG**	195.A NH1	PHE	110.A O	3.323
**ARG**	195.A NH2	GLN	234.A OE1	2.234
**GLU**	13.B N	GLY	44. A O	2.033
**LYS**	44.B NZ	PRO	129.A O	2.292
**THR**	109.B OG1	SER	125.A OG	2.736
**ASN**	113.B ND2	GLY	138.A O	3.071
**TYR**	10.A OH	SER	9.A N	2.488
**TYR**	10.A OH	SER	9.A OG	1.561
**ASP**	72.A OD1	SER	18.A OG	2.464
**ASP**	72.A OD2	SER	19.A N	3.105
**LEU**	81.A O	VAL	22.A N	2.898
**LYS**	7.A O	SER	23.A OG	3.271
**TYR**	10.A OH	SER	23.A OG	3.075
**GLU**	21.A O	SER	23.A OG	2.527

**THR**	109.A O	CYS	24.A SG	2.033
**LYS**	7.A O	LYS	25.A N	2.894
**LYS**	87.A O	SER	37.A OG	3.137
**SER**	16.B OG	GLY	44.A N	2.942
**GLU**	13.A O	MET	50.A N	2.559
**GLU**	42.A O	SER	58.A OG	2.300
**GLU**	42.A OE1	SER	58.A OG	3.242
**GLN**	82.A O	THR	71.A N	3.057
**TYR**	63.A O	THR	71.A OG1	3.534
**ASP**	8.A OD1	THR	80.A OG1	3.345
**LYS**	106.A O	TYR	82.A OH	2.814
**LYS**	7.B O	THR	93.A OG1	2.504
**GLU**	21.B O	THR	93.A OG1	3.225
**THR**	109.A O	CYS	98.A SG	4.030
**LYS**	111.A O	ALA	99.A N	1.638
**THR**	4.B O	THR	120.A OG1	2.482
**ARG**	107.B O	SER	122.A OG	2.848
**ILE**	108.B O	SER	123.A OG	2.872
**ASP**	90.B O	THR	126.A OG1	1.618
**VAL**	12.B O	LYS	127.A NZ	1.927
**ASN**	113.B O	LYS	127.A NZ	2.222
**CYS**	191.A O	GLN	151.A NE2	3.485
**GLU**	205.A O	SER	154.A OG	2.979
**GLU**	205.A OE2	THR	155.A OG1	1.670
**ASP**	153.A O	SER	159.A N	2.935
**LEU**	157.A O	THR	165.A OG1	3.012
**ILE**	148.A O	ILE	166.A N	1.322
**VAL**	175.A O	CYS	168.A SG	3.785
**THR**	164.A OG1	SER	173.A OG	2.877
**THR**	163.A O	SER	175.A OG	2.984
**THR**	164.A O	TRP	177.A N	2.518
**ALA**	114.A O	TYR	181.A OH	3.337
**GLU**	199.A OE2	LYS	190.A NZ	3.302
**THR**	163.A O	SER	212.A OG	2.642
**CYS**	137.A O	PHE	216.A N	3.214
**THR**	161.A OG1	THR	217.A N	2.750
**VAL**	147.A O	THR	217.A OG1	2.126
**THR**	161.A OG1	THR	217.A OG1	3.099
**TYR**	116.A O	CYS	233.A SG	2.986
**PHE**	173.A O	TYR	236.A N	2.665
**GLU**	140.A OE1	THR	242.A OG1	2.194
**CYS**	191.A O	THR	247.A OG1	2.601

Figure S1: SDS-PAGE confirmation using silver stain. The supernatant of recombinant CHO cells extract was collected and analyzed for accurate orientation. Panel (A) shows the supernatant results in reduced and non-reduced form. In non-reduced samples, many bands were visualized as 23kDa for two light chains, 50kDa for two heavy chain fragments. The white milky bands in 2-6 in non-reduced samples showed full-length antibody of approximately 150-170 kDa band size. Panel (B) indicates the purification of supernatant through Protein A resin column. M: molecular marker, P: supernatant before loading, PW: protein extract from the column, W1-W4: washing filtrates and E1-E4: Eluted antibody after washing. (+) indicate single immunoglobulin heavy and light fragment. (++) indicate heavy fragments with two heavy chains. (+++) mark shows a full length purified antibody of approximately 150-170kDa band size. 12 % gel was used for sample loading.

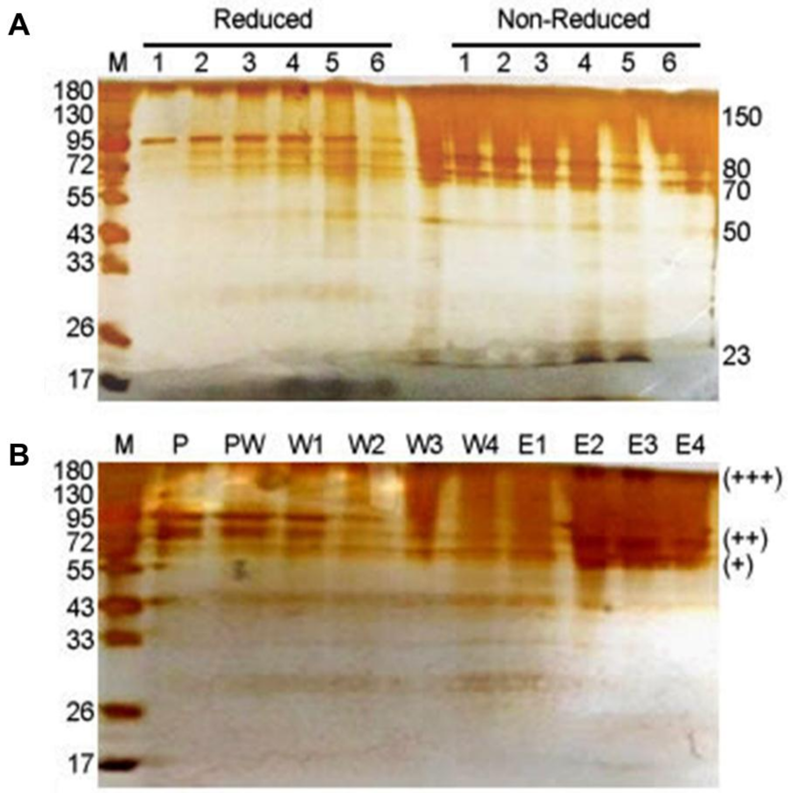

Supplement: Supplementary file 1 [file DataSheet_1.pdf]
